# The ecology of medical care in Korea: the association of a regular doctor and medical care utilization

**DOI:** 10.1186/s12913-022-08821-6

**Published:** 2022-11-28

**Authors:** Jeehye Lee, Yong-jun Choi, Dong-Hee Ryu

**Affiliations:** 1 National Emergency Medical Center, National Medical Center, Seoul, Korea; 2 Department of Social and Preventive Medicine, College of Medicine and Health Services Research Center, Hallym University, Chuncheon, Korea; 3 Institute of Social Medicine, College of Medicine, Hallym University, Chuncheon, Korea; 4 Department of Preventive Medicine, Daegu Catholic University School of Medicine, 33 Duryugongwon-Ro 17-Gil, Daegu, 42472 Nam-Gu Korea

**Keywords:** Phycisians, Primary care, Primary health care, Patient acceptance of health care

## Abstract

**Backgrounds:**

There is no registered regular doctor system in Korea, but people voluntarily select regular doctors for medical services. This study aims to study the ecology of medical care in Korea according to the presence and type of a regular doctor.

**Methods:**

This study was conducted using Korean Health Panel survey in 2018. The proportion of people who had health problems and received medical care in various settings was analyzed per 1,000 people according to the following groups: without a regular doctor, having a clinic physician as a regular doctor, and having a hospital physician as a regular doctor. The adjusted odds ratio for usage of medical services was calculated.

**Results:**

Health problems and medical care use increased in the groups in this order: group without a regular doctor, group with a clinic physician as a regular doctor, the group with a hospital physician a regular doctor. Having a hospital physician as a regular doctor was associated with higher odds of inpatient care and emergency room visits, and having a clinic physician as a regular doctor was not associated with odds of inpatient care and emergency room visits when adjusting demographic and health-related variables.

**Conclusion:**

Depending on whether having a regular doctor and a regular doctor’s type, different ecology of medical care was observed. The position and role of a regular doctor in the context of the Korea health care system should be considered from the perspective of primary care.

## Introduction

Primary care plays a significant role as the backbone of a national medical system [1]. The Institute of Medicine in United States defined primary care as “the provision of integrated, accessible health care services by clinicians who are accountable for addressing a large majority of personal health care needs, developing a sustained partnership with patients, and practicing in the context of family and community” [2]. South Korea has a national health insurance system that compensates medical services under a fee-for-service system; however, its primary care is fragile [3, 4]. Under its compensation system, essential features of primary care, including health behavior interventions and care coordination, are not adequately reimbursed. Primary care also lacks a gatekeeping function, and obtaining referral slips from a primary care physician is easy, which eventually allows patients to choose and visit clinics or hospitals freely [5]. Meanwhile, the public is using medical care by voluntarily selecting usual sources of care (USC), who has a regular doctor is about 20% of the population. Of the attributes of primary care, regular doctors are expected to implement comprehensiveness [6] and continuity based on physician and patient relationships [7].

Many studies [8–12] have found that USC was associated with lower medical costs, good health outcomes, and positive experiences. It was previously proved that in Korea, if patients with a chronic disease had a USC, they were less likely to visit the emergency room and use inpatient and outpatient care [13, 14]. The associated medical expenses were lower than for the people without a USC [13–15]. However, such results were obtained from a group of selected individuals. Since primary care is a crucial element in the health care system of the population, related research should be designed from the perspective of the population.

The ecology of the medical care model developed by White et al. in 1961 described the needs and demands of health care from a population perspective [16]. This framework sparked discussions on appropriate functions of primary care and medical education [17]. Further studies applying this model had been conducted in other countries, including South Korea [18]. This study aimed to describe the ecology of medical care in Korea using nationally representative data. The focus of this study was to examine the association between whether having a regular doctor and type of the doctor and the pattern of medical care utilization.

## Materials and methods

### Study design

It is a cross-sectional study analyzing the 2018 Korea Health Panel (KHP) survey data.

### Data

The Korea Health Panel (KHP) is a nationally representative annual survey which is jointly conducted by the National Health Insurance Corporation and the Korea Institute for Health and Social Affairs to produce basic data on health status, associated medical expenditures, and health behaviors [19]. The KHP survey uses Population and Housing Census data as its sampling frame [19]. Sample households were selected in the first step by extracting sample districts (cluster), in the second step by extracting sample households in sample districts, which is 2-stage cluster sampling method with probabilities proportion [19]. Households and household members living in 16 cities and provinces are subject to the survey, and 455 survey districts for the original sample are subject to 10,500 households [19]. The KHP is divided into the contents of the household survey and the individual survey of individual household members [19]. Households are investigated for demographic, socioeconomic characteristics, income and expenditure, contents of medical services used by household members, purchase costs of over-the-count drugs, and private medical insurance, and individual household members are investigated for chronic disease management, health behaviors, and medical care accessibility [19]. Questions other than medical care utilization, such as patient experience or activity restrictions, medical care accessibility, and a usual source of care, are asked in additional questions in appendix survey section [19].

### Study population

#### Group classification

The appendix survey of the KHP asks the following questions:” Do you have a usual source of care (medical institution)?” (Yes/No), “What types of medical institution do you usually go?” (Public Health Center/Clinic/Hospital/General Hospital/Tertiary Hospital/Oriental Clinic/Hospital/Etc.),” Do you have a regular doctor that you usually visit when you get sick or for medical examination/treatment/consultation?” (Yes/No). Two aspects of a participant’s medical care utilization were considered for group classification: whether the individual had a regular doctor and whether the individual had a medical institution to regularly visit or consult the doctor for any medical care. Based on the responses for two survey questions included in the appendix survey of KHP, “Do you have a doctor that you usually visit when you get sick or for medical examination/treatment/consultation?” and “What types of medical institution do you usually go?”, study participants were classified into three different groups: a group without a regular doctor, a group with a clinic physician as a regular doctor, and a group with a hospital physician as a regular doctor. According to this classification, people having a usual source of care without a regular doctor were classified as a group who do not have a regular doctor.

#### Inclusion and exclusion criteria

**Fig. 1:**
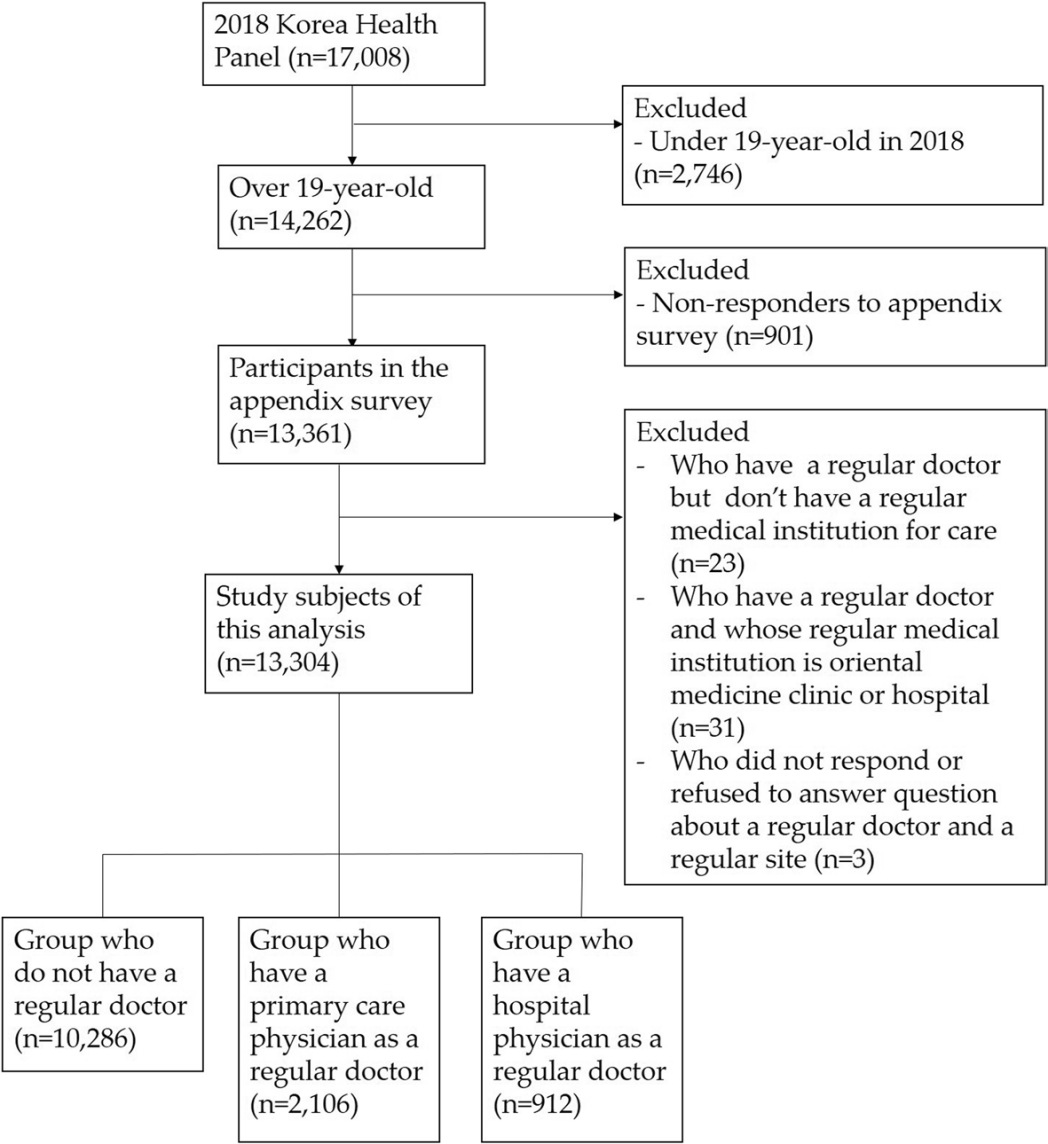
Selection process of the study subject

This study analyzed data from 14,262 KHP participants who were aged 19 years or above in 2018. Among these, 901 people were excluded from the analysis because they did not respond to the appendix survey. Those who reported having a regular doctor but did not specify the types of medical institution they usually visit or reported visiting oriental medicine hospitals as the regular source of care were also excluded from the analysis. Finally, the total number of study participants included in the analyses was 13,304 (Fig. 1).

### Variables

#### Covariates

Covariates included age (19–44, 45–64, and ≥ 65 years), household income (1st quintile (poorest) -5th quintile (wealthiest), and residential areas (urban and rural). For risk adjustments, Charlson Comorbidity Index (CCI), chronic diseases and self-rated health (SRH) were used. CCI was calculated and used as a continuous variable in the multivariate analyses based on doctor-diagnosed diseases. Likert-scale SRH was further classified into three groups: good (very good and good), fair, and bad (poor and very poor).

### Outcome variables

#### Health problems

In order to determine a person’s health problems, the following four aspects were considered: whether the person 1) was diagnosed with any chronic disease by a doctor, 2) had to be bed-ridden for most of the day due to illness or injury within the past month, 3) was absent from work or school due to illness or injury within the past month, and 4) ever felt that it was mentally or physically challenging to handle one’s own life within the past month. People who reported any of these above issues were categorized as those with health problems.

#### Medical care utilization

For ambulatory care and outpatient department visits, health care settings were classified into clinics, secondary hospitals, and tertiary hospitals. In addition, inpatient care, and emergency department visits were included. In this study, a secondary hospital was defined as a 'hospital' under the Medical Service Act, and refers to a medical institution with 30 to 99 beds. A tertiary hospital included a ‘general hospital’ (100 and more beds) and ‘tertiary general hospital’ (300 and more beds) according to the Medical Service Act. Additionally, tertiary general hospitals require a referral slip.

In inpatient care, medical institutions were classified into secondary hospital and tertiary hospital. For the analysis of inpatient care, only hospital-based settings were considered.

### Statistical analyses

The number of people per 1,000 with health problems and those who utilized medical care was estimated. The number of months of medical use experience for each individual was calculated. Medical use for each group was obtained by calculating person-month based on annual data. All person-month medical use of the study subjects is summed up. The above figure was divided by 12 months and then multiplied by the weight of the household members. The sum of the figures divided by the population aged 19 or older, and multiplied by 1,000. Separate analyses were conducted according to sex. A generalized estimating equations (GEE) procedure was performed to estimate the adjusted odds ratio (aOR), and demographics and health-related variables were controlled. In order to obtain aOR, each individual's medical use was obtained every month, and GEE analysis was performed by considering it as repeated measurement data for the individual. Cross-sectional weights for the sampled population were applied. SAS version 9.4 (SAS Institute Inc., Cary, NC, USA) was used for the analysis, and *p*-values < 0.05 indicated statistical significance (chi-square test).

### Ethics statement

Ethical evaluation was exempted by the institutional review board of Daegu Catholic University Medical Center (CR-22–007-PRO-001-R).

## Results

### The ecology of medical care in 2018

**Fig. 2:**
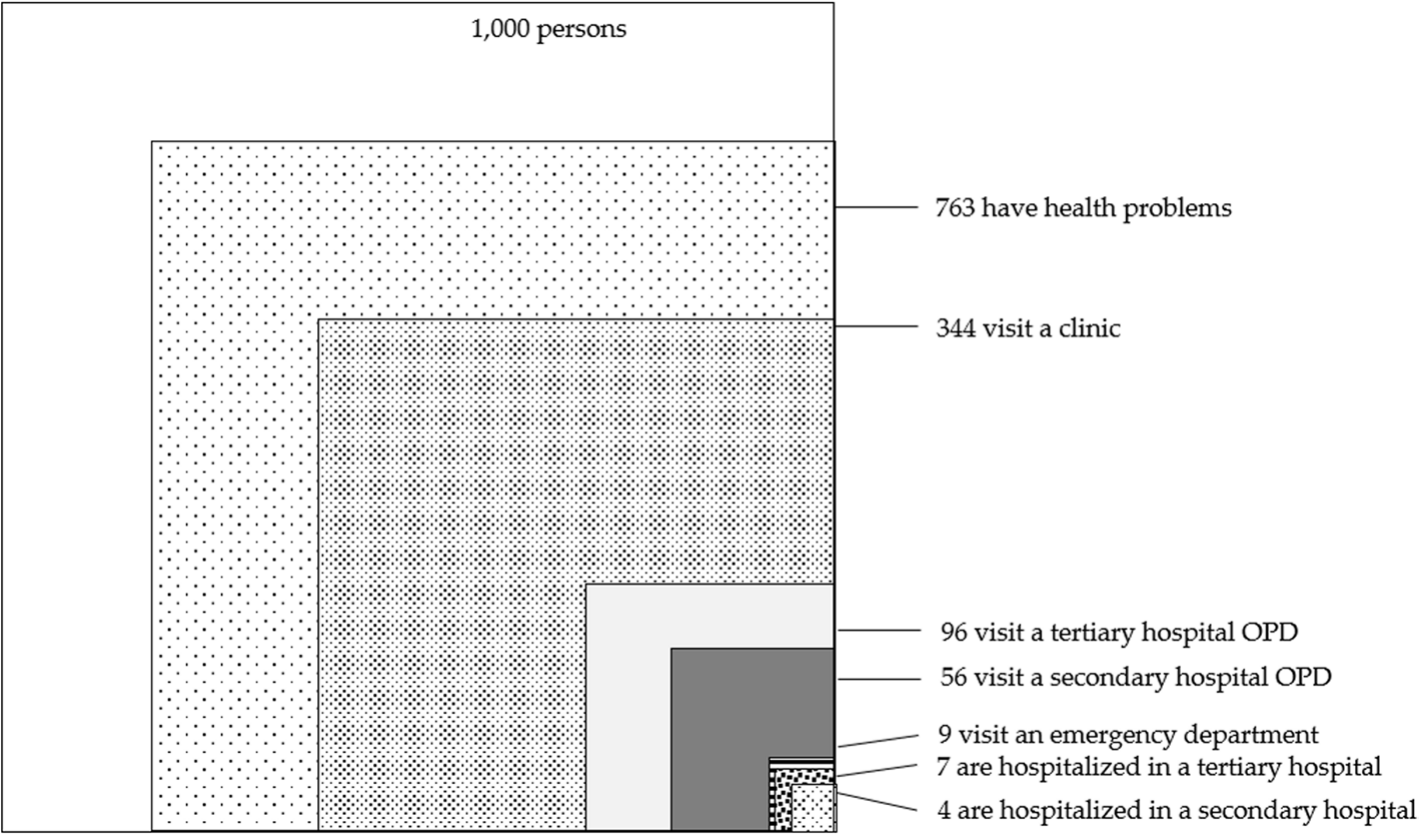
Monthly prevalence estimates of health problems and health care utilization in 2018 Korean population

Figure 2 illustrates estimated number of people per 1,000 residents aged 19 and over who had any health problem and/or medical care in the different care settings in an average month of 2018. Each square is not subset of the larger square.

### Demographics of study subjects by type and location of a regular doctor, according to sex

Table 1 shows demographics of study subjects by type and location of a regular doctor, according to sex. Men were less likely to have a regular doctor than women (No regular doctor-Men: 80.08%, Women: 74.96%). For both sexes, the percentage with a regular doctor increased with increasing age (19–44 in men: 7.59%, 65 and more in men: 34.28%, 19–44 in women: 12.22%, 65 and more in women: 37.41%). Residential area had no statistically significant difference in having a regular doctor and regular site. The higher the household income, the lower the percentage of having a regular doctor in both sexes. For women, the percentage without a regular doctor increased consistently as the household income was higher, with 66.61% in the 1st quintile and 80.61% in the 5th quintile not having a regular doctor. For men, 72.43% of the 1st quintile did not have a regular doctor, and 84.02% of the 4th quintile and 82.86% of the 5th quintile did not have a regular doctor. The worse the health condition, the higher the percentage with a regular doctor. In the group with 0 CCI, 82.59% of men and 79.7% of women did not have a regular doctor, whereas in the group with 2 or higher CCI, 59.54% of men and 57.36% of women did not have a regular doctor. In group whose CCI is 2 or higher, 23.8% of men and 21% of women had a hospital physician as a regular doctor. For self-rated health, in the group with good health, 84.6% of men and 81.77% of women had no regular doctor. In the group with bed health, 75.46% of men and 68.28% of women had no regular doctor. In men who answered that they were in poor health, 13.59% of them had a primary care physician as a regular doctor, and 10.95% of them had a hospital physician as a regular doctor. In women who answered that they were in poor health, 20.4% of them had a primary care physician as a regular doctor, and 11.32% of them had a hospital physician as a regular doctor. Those who have chronic disease are more likely to have a regular doctor.

**Table 1:** Demographics of study subjects by sex

	**Men**				**Women**			
	**No regular doctor**	**Regular doctor-Clinic physician**	**Regular doctor-Hospital physician**	***P* value**	**No regular doctor**	**Regular doctor-Clinic physician**	**Regular doctor-Hospital physician**	***P* value**
	4900 (80.08)	802 (13.11)	417 (6.81)		5386 (74.96)	1304 (18.15)	495 (6.89)	
Age								
19–44	1875 (92.41)	102 (5.03)	52 (2.56)	< .0001	1926 (87.78)	193 (8.80)	75 (3.42)	< .0001
45–64	1888 (80.00)	310 (13.14)	162 (6.86)		1959 (75.55)	443 (17.08)	191 (7.37)	
65 and more	1137 (65.72)	390 (22.54)	203 (11.73)		1501 (62.59)	668 (27.86)	229 (9.55)	
Area								
Urban	3825 (80.65)	604 (12.73)	314 (6.62)	0.12	4106 (75.23)	963 (17.64)	389 (7.13)	0.07
Rural	1075 (78.13)	198 (14.39)	103 (7.49)		1280 (74.12)	341 (19.75)	106 (6.14)	
Household income								
1^st^	494 (72.43)	122 (17.89)	66 (9.68)	< .0001	784 (66.61)	302 (25.66)	91 (7.73)	< .0001
2^nd^	794 (74.76)	168 (15.82)	100 (9.42)		938 (69.07)	299 (22.02)	121 (8.91)	
3^rd^	1046 (80.52)	180 (13.86)	73 (5.62)		1114 (75.27)	272 (18.38)	94 (6.35)	
4^th^	1251 (84.02)	160 (10.75)	78 (5.24)		1286 (80.27)	229 (14.29)	87 (5.43)	
5^th^	1315 (82.86)	172 (10.84)	100 (6.3)		1264 (80.61)	202 (12.88)	102 (6.51)	
Charlson Comorbidity Index								
0	3933 (85.59)	514 (11.19)	148 (3.22)	< .0001	4421 (79.70)	888 (16.01)	238 (4.29)	< .0001
1	627 (65.79)	193 (20.25)	133 (13.96)		618 (59.83)	286 (27.69)	129 (12.49)	
2 and more	340 (59.54)	95 (16.64)	136 (23.82)		347 (57.36)	130 (21.49)	128 (21.16)	
Chronic disease								
No	2886 (87.53)	298 (9.04)	113 (3.43)	< .0001	2556 (82.99)	404 (13.12)	120 (3.90)	< .0001
Yes	2014 (71.37)	504 (17.86)	304 (10.78)		2830 (68.94)	900 (21.92)	375 (9.14)	
Self-rated health								
Good	2154 (84.6)	286 (11.23)	106 (4.16)	< .0001	1996 (81.77)	337 (13.81)	108 (4.42)	< .0001
Fair	1974 (77.41)	377 (14.78)	199 (7.80)		2359 (72.94)	659 (20.38)	216 (6.68)	
Bad	772 (75.46)	139 (13.59)	112 (10.95)		1031 (68.28)	308 (20.40)	171 (11.32)	

Values are presented as number (%). A chi square test was performed to obtain the *p* value

### Ecology of medical care by having a regular doctor and type of the doctor, according to sex

Table 2 shows estimated number of people who had any health problem and/or use medical care in an average month per 1000 residents aged 19 and over by having a regular doctor and type of the doctor, according to sex. On a monthly basis, the estimated number of people with health problems per 1000 individuals increased in the following order: group without a regular doctor, group having a clinic physician as a regular doctor, group having a hospital physician as a regular doctor. For ambulatory care and outpatient department visits, those who have a clinic physician as a regular doctor were more likely to visit clinics than other groups. In contrast, individuals with a hospital physician as a regular doctor were more likely to visit secondary and tertiary hospitals than other groups. For inpatient care, the group having a hospital physician as a regular doctor had 2–3 times more instances of hospitalization in secondary and tertiary hospitals than other groups. For emergency department visits, the group having a hospital physician as a regular doctor had more instances of visit emergency department than other groups.

**Table 2:** Monthly prevalence estimates of health problems and health care utilization according to type and location of a regular doctor by sex

			**Men**		**Women**		
		**No regular doctor**	**Regular doctor-Clinic physician**	**Regular doctor-Hospital physician**	**No regular doctor**	**Regular doctor-Clinic physician**	**Regular doctor-Hospital physician**
**Health problems**		692.5 (676.7, 708.3)	918.1 (893.3, 942.8)	938.9 (906.7, 971.2)	754.3 (740.0, 768.6)	904.0 (881.9, 926.1)	937.0 (905.2, 968.7)
**Ambulatory Care or outpatient department visit**	**Clinic**	239.9 (231.0, 248.8)	604.8 (576.6, 632.9)	288.1 (253.81, 322.41)	356.3 (346.8, 365.8)	615.2 (593.9, 636.5)	389.4 (360.1, 418.8)
	**Secondary Hospital**	37.7 (34.1, 41.4)	52.2 (42.4, 61.9)	106.4 (83.0, 129.9)	62.0 (57.7, 66.2)	66.4 (56.7, 76.1)	132.8 (107.8, 157.8)
	**Tertiary Hospital**	68.5 (63.8, 73.2)	100.6 (84.8, 116.4)	372.2 (337.1, 407.2)	83.5 (78.7, 88.4)	82.2 (73.0, 91.4)	350.3 (317.9, 382.6)
**Hospitalization**	**Secondary Hospital**	3.0 (2.5, 3.6)	3.4 (2.0, 4.8)	5.4 (2.9, 7.9)	4.8 (4.1, 5.4)	5.4 (3.8, 6.9)	9.9 (5.4, 14.4)
	**Tertiary Hospital**	6.0 (5.1, 6.9)	9.4 (6.2, 12.5)	23.5 (16.8, 30.3)	5.9 (5.1, 6.7)	6.3 (4.6, 8.0)	21.1 (14.9, 27.2)
**Emergency Department Visit**		7.20 (6.4, 8.0)	10.8 (8.0, 13.5)	17.4 (12.8, 22.0)	8.2 (7.3, 9.1)	8.5 (6.8, 10.2)	16.4 (12.0, 20.8)

Values are presented as number per 1,000 (95% confidence interval)

### Adjusted odds ratio of medical care utilization in the different care setting in 2018 according to sex

Tables 3 and 4 shows aOR of medical care utilization in the different care setting in 2018. The aOR (95% confidence interval) for ambulatory care visits in the groups having a clinic physician and a hospital physician as a regular doctor were 3.39 (2.98, 3.86), 0.65 (0.54, 0.79) and 2.16 (1.96, 2.37), 0.72 (0.62, 0.85) in men and women, respectively. In outpatient department visits in tertiary hospitals, the aOR in the group having a hospital physician as a regular doctor was 3.86 (3.16, 4.71) in men and 3.22 (2.76, 3.76) in women. The aOR of inpatient care in tertiary hospitals in the group having a hospital physician as a regular doctor was 1.59 (1.10, 2.28) in men and 1.72 (1.22, 2.43) in women. The aOR of emergency room visit in the group having a hospital physician as a regular doctor was 1.56 (1.11, 2.19) in men and 1.46 (1.06, 2.01) in women.

**Table 3:** Adjusted odds ratio of medical care utilization-Men

	Ambulatory Care			Inpatient Care		Emergency Department Visit
	Clinic	Hospital	General, Tertiary Hospital	Hospital	General, Tertiary Hospital	
Age group						
19–44	0.31 (0.27, 0.35)	0.53 (0.41, 0.70)	0.45 (0.35, 0.57)	0.37 (0.21, 0.64)	0.60 (0.38, 0.97)	0.83 (0.61, 1.14)
45–64	0.54 (0.48, 0.61)	0.93 (0.76, 1.15)	0.58 (0.50, 0.69)	0.61 (0.43, 0.88)	0.63 (0.45, 0.88)	0.79 (0.63, 1.01)
65 and more	1.00 (REF)	1.00 (REF)	1.00 (REF)	1.00 (REF)	1.00 (REF)	1.00 (REF)
Household income						
1^st^	0.97 (0.82, 1.16)	1.15 (0.82, 1.62)	1.09 (0.84, 1.41)	1.09 (0.65, 1.83)	1.42 (0.91, 2.20)	1.26 (0.89, 1.78)
2^nd^	1.04 (0.91, 1.19)	0.98 (0.76, 1.27)	1.00 (0.82, 1.23)	0.85 (0.51, 1.39)	1.63 (1.12, 2.39)	1.41 (1.05, 1.89)
3^rd^	1.04 (0.92, 1.18)	0.95 (0.72, 1.24)	1.01 (0.84, 1.22)	0.80 (0.48, 1.31)	1.67 (1.15, 2.44)	1.10 (0.81, 1.48)
4^th^	1.08 (0.96, 1.22)	1.02 (0.79, 1.30)	0.92 (0.76, 1.11)	0.90 (0.56, 1.47)	1.27 (0.85, 1.91)	1.18 (0.88, 1.58)
5^th^	1.00 (REF)	1.00 (REF)	1.00 (REF)	1.00 (REF)	1.00 (REF)	1.00 (REF)
Area						
Urban	0.99 (0.90, 1.07)	0.87 (0.73, 1.03)	0.96 (0.85, 1.09)	0.90 (0.65, 1.23)	0.89 (0.69, 1.14)	0.81 (0.67, 0.99)
Rural	1.00 (REF)	1.00 (REF)	1.00 (REF)	1.00 (REF)	1.00 (REF)	1.00 (REF)
Charlson Comorbidity Index						
0	0.76 (0.64, 0.92)	0.90 (0.67, 1.22)	0.27 (0.22, 0.34)	0.84 (0.54, 1.32)	0.25 (0.16, 0.37)	0.56 (0.41, 0.76)
1	1.28 (1.05, 1.55)	1.37 (1.03, 1.83)	0.57 (0.46, 0.70)	1.20 (0.74, 1.94)	0.41 (0.29, 0.59)	0.57 (0.41, 0.78)
2 = <	1.00 (REF)	1.00 (REF)	1.00 (REF)	1.00 (REF)	1.00 (REF)	1.00 (REF)
Chronic diseases						
No	0.52 (0.47, 0.57)	0.59 (0.48, 0.74)	0.50 (0.43, 0.58)	0.75 (0.51, 1.10)	0.63 (0.47, 0.86)	0.71 (0.57, 0.89)
Yes	1.00 (REF)	1.00 (REF)	1.00 (REF)	1.00 (REF)	1.00 (REF)	1.00 (REF)
Self-rated health						
Good	1.00 (REF)	1.00 (REF)	1.00 (REF)	1.00 (REF)	1.00 (REF)	1.00 (REF)
Fair	1.26 (1.14, 1.39)	1.19 (0.98, 1.45)	1.37 (1.18, 1.59)	1.36 (0.90, 2.05)	1.20 (0.88, 1.64)	1.29 (1.02, 1.65)
Bad	1.12 (0.97, 1.29)	1.67 (1.29, 2.16)	1.95 (1.62, 2.35)	2.52 (1.66, 3.82)	2.37 (1.73, 3.25)	1.92 (1.50, 2.47)
Regular doctor						
No regular doctor	1.00 (REF)	1.00 (REF)	1.00 (REF)	1.00 (REF)	1.00 (REF)	1.00 (REF)
Regular doctor-Clinic physician	3.39 (2.98, 3.86)	1.01 (0.81, 1.26)	0.91 (0.75, 1.12)	0.77 (0.49, 1.21)	1.04 (0.72, 1.52)	1.23 (0.91, 1.65)
Regular doctor-Hospital physician	0.65 (0.54, 0.79)	1.89 (1.40, 2.56)	3.86 (3.16, 4.71)	1.04 (0.63, 1.71)	1.59 (1.10, 2.28)	1.56 (1.11, 2.19)

Values are presented as adjusted odds ratios (95% confidence intervals). Generelized estimating equation. Cross-sectional weights for the sampled population were applied

**Table 4:** Adjusted odds ratio of medical care utilization-Women

	Ambulatory Care			Inpatient Care		Emergency Department Visit
	Clinic	Hospital	General, Tertiary Hospital	Hospital	General, Tertiary Hospital	
Age group						
19–44	0.36 (0.32, 0.40)	0.80 (0.64, 0.99)	0.53 (0.44, 0.65)	1.10 (0.69, 1.75)	0.56 (0.37, 0.85)	0.75 (0.56, 0.99)
45–64	0.57 (0.52, 0.64)	1.03 (0.86, 1.23)	0.81 (0.70, 0.93)	1.18 (0.81, 1.71)	0.96 (0.72, 1.28)	0.76 (0.60, 0.96)
65 and more	1.00 (REF)	1.00 (REF)	1.00 (REF)	1.00 (REF)	1.00 (REF)	1.00 (REF)
Household income						
1^st^	1.28 (1.11, 1.49)	1.34 (1.06, 1.70)	0.95 (0.77, 1.16)	1.75 (1.08, 2.85)	0.97 (0.68, 1.39)	0.90 (0.66, 1.22)
2^nd^	1.09 (0.97, 1.22)	1.18 (0.96, 1.45)	1.01 (0.86, 1.20)	1.22 (0.81, 1.83)	0.96 (0.68, 1.36)	1.00 (0.76, 1.32)
3^rd^	1.11 (1.00, 1.24)	1.12 (0.91, 1.37)	0.91 (0.77, 1.07)	1.05 (0.71, 1.56)	0.93 (0.65, 1.32)	0.92 (0.69, 1.24)
4^th^	1.04 (0.94, 1.16)	1.05 (0.86, 1.28)	0.87 (0.74, 1.02)	0.99 (0.66, 1.51)	0.90 (0.59, 1.37)	1.00 (0.74, 1.34)
5^th^	1.00 (REF)	1.00 (REF)	1.00 (REF)	1.00 (REF)	1.00 (REF)	1.00 (REF)
Area						
Urban	0.99 (0.92, 1.06)	0.91 (0.80, 1.04)	0.96 (0.86, 1.06)	0.86 (0.67, 1.10)	1.02 (0.81, 1.27)	0.86 (0.72, 1.04)
Rural	1.00 (REF)	1.00 (REF)	1.00 (REF)	1.00 (REF)	1.00 (REF)	1.00 (REF)
Charlson Comorbidity Index						
0	1.12 (0.96, 1.31)	1.21 (0.96, 1.54)	0.29 (0.25, 0.34)	0.89 (0.59, 1.35)	0.30 (0.22, 0.41)	0.75 (0.58, 0.97)
1	1.76 (1.47, 2.09)	1.38 (1.06, 1.80)	0.56 (0.48, 0.67)	0.99 (0.62, 1.57)	0.51 (0.36, 0.74)	0.76 (0.56, 1.05)
2 = <	1.00 (REF)	1.00 (REF)	1.00 (REF)	1.00 (REF)	1.00 (REF)	1.00 (REF)
Chronic diseases						
No	0.59 (0.55, 0.64)	0.69 (0.59, 0.80)	0.54 (0.48, 0.62)	0.63 (0.47, 0.83)	0.76 (0.58, 1.01)	0.80 (0.64, 1.00)
Yes	1.00 (REF)	1.00 (REF)	1.00 (REF)	1.00 (REF)	1.00 (REF)	1.00 (REF)
Self-rated health						
Good	1.00 (REF)	1.00 (REF)	1.00 (REF)	1.00 (REF)	1.00 (REF)	1.00 (REF)
Fair	1.33 (1.23, 1.44)	1.32 (1.13, 1.53)	1.26 (1.11, 1.44)	1.29 (0.95, 1.75)	1.34 (0.98, 1.82)	1.17 (0.92, 1.50)
Bad	1.41 (1.25, 1.59)	1.90 (1.57, 2.29)	1.79 (1.54, 2.08)	2.78 (2.02, 3.84)	2.88 (2.08, 3.99)	2.34 (1.80, 3.05)
Regular doctor						
No regular doctor	1.00 (REF)	1.00 (REF)	1.00 (REF)	1.00 (REF)	1.00 (REF)	1.00 (REF)
Regular doctor-Clinic physician	2.16 (1.96, 2.37)	0.90 (0.75, 1.08)	0.67 (0.59, 0.77)	0.94 (0.68, 1.31)	0.77 (0.58, 1.03)	0.90 (0.71, 1.13)
Regular doctor-Hospital physician	0.72 (0.62, 0.85)	1.83 (1.41, 2.36)	3.22 (2.76, 3.76)	1.48 (0.89, 2.49)	1.72 (1.22, 2.43)	1.46 (1.06, 2.01)

Values are presented as adjusted odds ratios (95% confidence intervals). Generelized estimating equation. Cross-sectional weights for the sampled population were applied

## Discussion

The ecology of medical care in Korea has been previously analyzed with the 2012 KHP data [18]. Compared to the previous study results, the overall outpatient care has increased, while inpatient care showed no notable changes. However, caution should be exercised when comparing the present results with the previous results for two reasons. First, this study's definition of a tertiary hospital differed from the previous one [18]. The present study defined 'tertiary hospital' by including general and tertiary hospitals. Second, while the previous study [18] considered five factors (physical and mental stress, frustration, unmet basic needs, job stress, and anxiety about the future), only physical and mental stress were used in the present study to define any mental health problem.

The proportion of having no regular doctors was higher than that of having a regular doctor. A higher percentage of having a regular doctor was associated with higher age, lower household income, higher CCI, having chronic diseases, and worse SRH. The proportion of women having a regular doctor was higher than that of men. The difference was due to a higher proportion of women having a clinic physician as a regular doctor. The difference in the retention rate of a regular doctor by the population group observed in this study is consistent with the results of previous studies [6, 20–22] that there is a correlation between demographic and health status and a USC (regular doctor). According to Andersen's behavioral model of health service use, a USC is classified as an enabling component along with insurance [23, 24]. However, in a universal health coverage system, especially under a fee-for-service payment system, USC could also be interpreted as a predisposing factor that is closer to attitude toward medical use. Considering that the group having hospital physicians as a regular doctor use medical services more than the group without a regular doctor, even after adjusting the various factors, the presence of a hospital physician as a regular doctor can be attributed to the preference for medical use in groups with USC in Korea.

Having a clinic physician as a regular doctor does not affect the odds of inpatient care and emergency room visits except for ambulatory care visits when adjusting demographic and health-related variables. Unlike previous studies, this study did not show the association of USC and lower rate of reducing emergency room visits [13, 14]. That may be because the subject of the study is different between this study and previous studies, and the functions and quality of clinics in Korea are very heterogeneous [25]. While this study targeted the entire population over the age of 19, the previous study conducted only those who had chronic diseases or those who answered that there was a demand for medical care use [13, 14]. The heterogeneity of primary medical institutions is due to the absence of the concept of primary care and the absence of training for primary care physicians [25]. Specialized physicians mostly provide medical services in primary care [26]. In addition, the quality management mechanism of primary medical institutions is insufficient [27].

In the group with hospital-physician as a regular doctor, there was an increase in emergency room visits or hospitalization, which was contrary to the expected results of a regular doctor. There are several reasons for having a hospital physician as a regular doctor: first, to use the hospital as a primary medical institution for accessibility in medically vulnerable areas. Second, to regularly visit a hospital doctor due to the severity of the disease. Third, the tendency to prefer medical care in hospitals [28] where multiple specialists work and multiple tests are available. In any case, if the hospital is used as a regular site for medical care, it will be easy to transfer to other specialized departments in the hospital so that number of tests and hospitalizations will increase. In other words, medical care use may also increase due to patients' preference for medical use or disease severity, but there would be more supply-induced care in hospital care settings. In this study, it is difficult to determine which factors increase medical care use for groups with a hospital physician as a regular doctor. It is necessary to consider whether usage of more medical services by this group lead to high quality medical care and good health outcomes.

Meanwhile, though this study adjusted factors related to health care utilization, the presence of an unmet need in a group without a regular doctor should be analyzed in the future. Most people did not have a regular doctor. Those who reported high CCI, presence of chronic disease, or poor SRH tended to be more likely to have a regular doctor. However, about 60% of those with a CCI of 2 or higher and 70% of those who reported poor SRH said they did not have a regular doctor. Many previous studies [24, 29–31] have indicated that USC improves healthcare access and USC reduces unmet medical care needs. Further research is needed to determine whether low medical care utilization in a group without a regular physician is related to unmet medical care needs.

This study has several limitations. First, as it is an ecological study, the reasons behind the association between the results and the presence of a regular doctor are unknown. Second, unobserved confounders, which could not be considered due to data restrictions, may exist. Third, presence of a hospital physician as a regular doctor is contradictory to the primary health care setting. Fourth, there could be bias because the CCI was measured in the year in which medical utilization was observed. Nevertheless, the strength of this study is that it shows the different patterns of medical care utilization for the entire population depending on the types of a USC. The framework of the ecology of medical care is relevant in this study as its focus is on the entire population. In addition, this model has been studied by several researchers over the years. The results of the analysis of the impact of having a regular doctor on health care utilization in South Korea using this model contributed to knowledge related to health care utilization.

## Conclusions

Health problems and medical care utilization increased in the groups in this order: group without a regular doctor, group with a clinic physician as a regular doctor, the group with a hospital physician a regular doctor. Having a hospital physician as a regular doctor was associated with higher odds of inpatient care and emergency room visit, and having a clinic physician as a regular doctor was not associated with odds of inpatient care and emergency room visits when adjusting demographic and health-related variables. Depending on whether having a regular doctor and a regular doctor’s type, different ecology of medical care is observed. The position and role of a regular doctor in the context of the Korea health care system should be considered from the perspective of primary care.

## Data Availability

The data presented in this study are openly available in [https://www.khp.re.kr:444/web/data/data.do].

## Abbreviations

KHP: Korea Health Panel
USC: Usual source of care
CCI: Charlson Comorbidity Index
SRH: Self-rated health
GEE: Generalized estimating equations
aOR: Adjusted odds ratio
